# Who engages in the arts in the United States? A comparison of several types of engagement using data from The General Social Survey

**DOI:** 10.1186/s12889-021-11263-0

**Published:** 2021-07-08

**Authors:** Jessica K. Bone, Feifei Bu, Meg E. Fluharty, Elise Paul, Jill K. Sonke, Daisy Fancourt

**Affiliations:** 1 Research Department of Behavioural Science and Health, Institute of Epidemiology & Health, University College London, London, UK; 2 Center for Arts in Medicine, University of Florida, Gainesville, Florida, USA

**Keywords:** Arts, Culture, Social gradient, Wellbeing, Health, United States

## Abstract

**Background:**

Engaging in the arts is a health-related behavior that may be influenced by social inequalities. While it is generally accepted that there is a social gradient in traditional arts and cultural activities, such as attending classical music performances and museums, previous studies of arts engagement in the US have not adequately investigated whether similar demographic and socioeconomic factors are related to other forms of arts engagement.

**Methods:**

Using cross-sectional data from the General Social Survey (GSS) in the US, we examined which demographic, socioeconomic, residential, and health factors were associated with attendance at arts events, participation in arts activities, membership of creative groups, and being interested in (but not attending) arts events. We combined data from 1993 to 2016 in four analytical samples with a sample size of 8684 for arts events, 4372 for arts activities, 4268 for creative groups, and 2061 for interested non-attendees. Data were analysed using logistic regression.

**Results:**

More education was associated with increased levels of all types of arts engagement. Parental education demonstrated a similar association. Being female, compared to male, was also consistently associated with higher levels of engagement. Attendance at arts events was lower in participants with lower income and social class, poorer health, and those living in less urban areas. However, these factors were not associated with participation in arts activities or creative groups or being an interested non-attendee.

**Conclusions:**

Overall, we found evidence for a social gradient in attendance at arts events, which was not as pronounced in participation in arts activities or creative groups or interest in arts events. Given the many benefits of engagement in the arts for education, health, and wider welfare, our findings demonstrate the importance of identifying factors to reduce barriers to participation in the arts across all groups in society.

**Supplementary Information:**

The online version contains supplementary material available at 10.1186/s12889-021-11263-0.

## Background

There are many known social inequalities in health, including differences in healthy life expectancy and mortality [1, 2]. These disparities may be partially explained by a social gradient in a variety of health behaviors, including diet, obesity, physical activity, alcohol consumption, and smoking [3–5]. Health behavior norms may be learnt within the socioeconomic context, with social determinants influencing behavior throughout the life course [6]. Engaging in the arts is an example of a health-related behavior that demonstrates social inequalities [7, 8].

Arts engagement typically refers to different types of creative activity, from actively participating in the arts (e.g. dancing, singing, acting, painting, reading) to more receptive cultural engagement (e.g. going to museums, galleries, exhibits, performances and the theater [9]). It can also encompass broader creative activities that, whilst not always labelled as ‘arts’, share similar properties of creative skill and imagination (e.g. gardening, cooking, and hobby or book groups [10]). In 2019, the World Health Organization identified more than 3000 studies showing the beneficial impact of arts engagement on mental and physical health and social determinants of health, from education to social cohesion and welfare [9]. Despite growing awareness of the benefits of engaging with the arts, there is a social gradient in arts participation. Previous surveys have found that arts engagement in the United States (US) may differ according to socioeconomic status, education, and income [11–13]. Similar factors are associated with inequalities in access to health care and health and social outcomes [14–17]. Varying engagement in the arts may therefore further contribute to health and social inequalities [8]. However, the literature on this topic is limited by a number of factors.

First, many previous studies have focused on certain demographic or socioeconomic predictors of arts engagement without always taking into account the broad range of factors that may be related to one another. From these studies, the most consistent predictors of increased arts engagement are higher levels of education and income [12, 13, 18–24]. There have been extensive efforts to differentiate the effects of education and income on arts engagement, and it appears that both independently contribute to engagement levels [21, 25]. However, education may be more strongly associated with attending highbrow cultural events, whereas income is more strongly associated with other forms of arts engagement [25]. Further, self-identified social class may be another important factor which should be studied alongside income and education [23]. There is also evidence for lower rates of engagement in Black than White racial/ethnic groups [12, 18, 22, 26, 27]. Still, it remains unclear whether race/ethnicity has a strong association with engagement after other factors, particularly education and income (as interconnected systems that contribute to structural racism), have been taken into account [18, 21, 22, 27, 28].

Additionally, there are other factors that could be associated with arts engagement that have not been investigated in the US to date. In the UK, there are geographical differences in participation independent of individual demographic and socio-economic backgrounds [29]. Further, living alone is associated with fewer perceived opportunities to engage in the arts and those with poorer physical and mental health may experience more barriers to engaging [30]. As many previous studies of arts engagement in the US are based on the Survey of Public Participation in the Arts (SPPA; National Endowment for the Arts), which does not collect data on physical and mental health, these factors have not been investigated.

Moreover, in the US, most research on predictors of arts engagement has measured engagement with ‘benchmark’ arts activities, as defined in the SPPA. These activities include attending jazz, classical music, opera, musical or non-musical plays, ballet performances, and art museums or art galleries. Although these activities are not intended to be comprehensive [31], they have repeatedly been used as a metric of engagement in the arts. This has led to the perception that arts participation is declining in the US [11, 22, 32]. However, when defined more broadly, including other types of arts activities and going beyond the non-profit sector to recognize the many diverse commercial forms of cultural expression, participation is not declining and the way in which people participate may instead be changing [13, 33, 34]. There may be a growing gap between arts participation metrics and the ways in which people participate, and this could be affecting our understanding of the predictors of engagement [35].

Therefore, in this study, we used a large nationally representative sample of adults in the US (the General Social Survey; GSS) to investigate predictors of different types of arts engagement. Specifically, we were interested in whether there are social inequalities in engagement in the arts, as found in other health-related behaviors. To do this, we tested which demographic, socioeconomic, residential, and health factors were associated with attendance at arts events, participation in arts activities, and membership of creative groups. Further, in order to differentiate between non-attendance due to a lack of interest versus non-attendance due to barriers or a lack of opportunities, we investigated whether similar factors were associated with being interested in, but not attending, arts events. Finally, we examined whether engagement changed across time, from 1993 to 2016, and whether associations between demographic and socioeconomic factors and engagement changed over these two decades.

## Methods

### Sample

Participants were drawn from the General Social Survey (GSS); a repeated cross-sectional and rotating panel study of adults aged 18 and over in the US [36]. Each survey year was an independently drawn sample of English-speaking individuals living in non-institutional arrangements. From 2006 onwards, Spanish-speakers were added to the target population. Full probability sampling was employed, and surveys sub-sampled non-respondents from 2004 onwards.

We used data from GSS waves at which arts outcomes were measured between 1993 and 2016. Each wave included a unique sample of individuals so we were able to combine data across waves. We used four indicators of arts engagement (arts events, arts activities, creative groups, and interested non-attendees), each measured in different waves of the GSS. Arts events were measured in 1993, 1998, 2002, 2012 and 2016, arts activities were measured in 1993, 1998, and 2002, creative groups were measured in 1993, 1994, 2004, and 2010, and interested non-attendees were measured in 2012 and 2016. We therefore identified four samples, one for each outcome. When combining samples across all relevant years, the total number of participants was 14,890, 7203, 12,311, and 7687 for arts events, activities, creative groups, and interested non-attendees respectively. We then restricted the sample just to participants with complete data on arts variables, which produced a final sample size of 8684 for arts events, 4372 for arts activities, 4268 for creative groups, and 2061 for interested non-attendees (see Supplementary Table 1 for further details).

All participants gave informed consent and this study has Institutional Review Board approval from the University of Florida (IRB201901792) and ethical approval from University College London Research Ethics Committee (project 18839/001).

### Arts engagement outcomes

#### Arts events

﻿Participants were asked whether they had attended arts events in the last 12 months, not including school performances. In 1993, attendance at three events was measured as the following: a) art museum or gallery, b) ballet or dance performance, and c) classical music or opera performance. In 1998 and 2002, two additional events were added to this list: d) popular music performance, and e) non-musical stage play performance. In 2012 and 2016, attendance at two types of event was measured; a) music, theatre, or dance performance, and b) art exhibit (including paintings, sculpture, textiles, graphic design, or photography). Due to these differences in measurement across years, we collapsed all responses into a binary variable indicating attendance at any event in the last 12 months (0 = none, 1 = one or more). As this does not entirely account for the changes in question style, we tested whether the changing definition of arts events altered our findings in sensitivity analyses (outlined below). For full details of the questions asked in each wave, see Supplementary Table 2.

#### Arts activities

Participants self-reported whether they participated in any kind of arts activity in the last 12 months, including: a) making art or craft objects, b) taking part in music, dance, or theatrical performance, and c) playing a musical instrument (Supplementary Table 2). This was coded as a binary variable (0 = none, 1 = one or more), and was measured consistently in 1993, 1998, and 2002.

#### Creative groups

Participants were asked about the groups or organizations of which they were a member in 1993, 1994, 2004, and 2010. The creative groups were hobby or garden clubs and literary, art, discussion, or study groups (Supplementary Table 2). A binary variable was created indicating membership in either of these group types (0 = none, 1 = one or more).

#### Interested non-attendees

In the 2012 and 2016 GSS, participants who responded to the arts event questions were also asked if there was an arts event during the last 12 months that they had wanted to go to but did not attend (0 = no, 1 = yes). In 2012, only participants who had not attended an event during the last 12 months were asked this question. In 2016, all participants who were asked about arts event attendance were also asked whether there was an event that they had wanted to go to but did not attend. As we aimed to include only participants who were interested non-attendees, we excluded those who reported attending an arts event in 2016 (*n* = 738 excluded).

### Exposures

We examined whether a range of demographic, socioeconomic, residential, and health factors were associated with arts engagement. Demographics included age (years), sex (male or female), race/ethnicity (White, Black, or Other) and marital status (married, separated/divorced/widowed, or never married). Socioeconomic factors included total number of years of education (0–20 years), parental years of education (highest reported maternal or paternal education; 0–20 years), employment status in the last week (employed, unemployed or not currently working, retired, keeping house, or other), family income in constant dollars (base = 1986; $0 to $9999, $10,000 to $19,999, $20,000 to $29,999, $30,000 to $49,999, or $50,000+), subjective satisfaction with financial situation (not satisfied at all, more or less satisfied, or pretty well satisfied), and a subjective rating of social class (lower class, working class, middle class, or upper class).

Residential factors included level of urbanicity (medium to large city with 50,000 people or more; suburb of a medium to large city; unincorporated area of a medium to large city; small city, town or village of 2500 to 50,000 people; and smaller areas or open country), number of people living in the household (1–10), and whether there was an area within a mile of their home where they would be afraid to walk alone at night (yes or no).

Finally, we included a general health rating (excellent, good, fair, or poor).

### Statistical analyses

We used four logistic regression models to test cross-sectional associations between demographic, socioeconomic, residential, and health exposures and binary arts engagement outcomes. Each outcome (arts events, arts activities, creative groups, interested non-attendees) was modelled separately. Where there was evidence of a non-linear association between age and arts engagement, we included a quadratic age term. As a number of similar exposures were included, multicollinearity was assessed to ensure that Variance Inflation Factors were less than 10 [37]. All analyses were weighted to account for the sub-sampling of non-respondents and the number of adults in the household using weights supplied by the GSS [36]. We accounted for clustering of participants within primary sampling units by using robust standard errors.

We also tested whether there was any evidence that associations between arts engagement outcomes and age, race/ethnicity, class, income, and sex differed over time. We included an interaction term between each exposure and survey year in separate logistic regression models. Where there was evidence for an interaction, we then examined the association between the exposure and arts engagement separately in each survey year.

For participants with missing data on exposures, we imputed data using multiple imputation by chained equations (MICE [38]). We used linear, logistic, ordinal, and multinomial regression and predictive mean matching according to variable type, generating 50 imputed data sets (maximum missing data ranged from 10 to 35% in each sample; Supplementary Table 3). The imputation model included all variables used in analyses, auxiliary variables, and the survey weights. Auxiliary variables were split ballot group, interviewer’s rating of the respondent’s attitude toward the interview and understanding of questions, respondent’s rating of their family income (relative to other Americans), and geographic mobility since age 16. Imputations were performed separately according to survey year. For creative groups, several exposures (satisfaction with financial situation, general health rating, and feeling afraid in neighborhood) and an auxiliary variable (relative income) were missing for all participants in some years so were not included in the imputations or analyses. All other variables were successfully imputed. The results of analyses did not vary between complete cases and imputed data sets (Supplementary Table 4), so findings from the imputed data are reported. All analyses were performed using Stata 16 [39].

#### Sensitivity analysis

We tested whether the changing definition of arts event attendance altered our findings. In this analysis, we used the most homogenous measures of arts events, those included from 1998 to 2016. We therefore repeated the main analysis excluding participants from 1993 (which used a narrower definition of arts events) and examined whether similar factors were associated with arts event attendance in this subsample (*n* = 7094; Supplementary Table 7).

## Results

### Arts events

In total, 8684 participants provided data on attendance at arts events, 53% of whom were female and 78% were White (Table 1). These participants ranged in age from 18 to 89 years, with a mean age of 46.6 (SD = 17.0). Overall, 56% had attended an arts event in the last 12 months, although this varied across years (1993: 48%, 1998: 62%, 2002: 66%, 2012: 46%, 2016: 50%).

**Table 1:** *Demographic characteristics of the samples, with data combined across all included survey years*

	Events *n* = 8684	Activities *n* = 4372	Groups *n* = 4268	Interested non-attendees *n* = 2061
	**Percentage**			
Female	53%	53%	56%	53%
Race/ethnicity				
White	78%	81%	81%	70%
Black	14%	12%	12%	20%
Other	8%	7%	7%	10%
Marital status				
Married	55%	56%	60%	50%
Separated/divorced/widowed	21%	21%	20%	25%
Never married	24%	23%	20%	25%
Employment status				
Employed	63%	65%	62%	57%
Unemployed/not working	6%	5%	6%	7%
Retired	15%	13%	14%	17%
Keeping house	10%	11%	12%	12%
Other	6%	6%	6%	7%
Family income				
$0–$9999	18%	17%	16%	28%
$10,000–$19,999	21%	21%	21%	24%
$20,000–$29,999	18%	20%	17%	16%
$30,000–$49,999	23%	21%	23%	21%
$50,000+	20%	21%	23%	11%
Satisfaction with financial situation				
Not satisfied at all	28%	27%	–	34%
More or less satisfied	44%	44%	–	44%
Pretty well satisfied	28%	29%	–	22%
Social class				
Lower class	7%	5%	6%	13%
Working class	46%	45%	43%	54%
Middle class	44%	46%	48%	32%
Upper class	3%	4%	3%	1%
General health rating				
Excellent	28%	32%	–	21%
Good	47%	47%	–	42%
Fair	19%	16%	–	28%
Poor	6%	5%	–	9%
Level of urbanicity				
Med-large city (50,000+)	31%	31%	29%	31%
Suburb	35%	36%	33%	29%
Unincorporated area	13%	9%	15%	18%
Small city or town	11%	14%	11%	9%
Smaller areas or country	10%	10%	12%	13%
Feels afraid in neighborhood	34%	38%	–	31%
**Mean (SE)**				
Age	46.61 (0.23)	44.80 (0.33)	45.92 (0.34)	49.14 (0.52)
Years of education	13.44 (0.05)	13.20 (0.07)	13.55 (0.06)	12.55 (0.11)
Parental years of education	12.07 (0.06)	11.85 (0.09)	12.11 (0.09)	11.26 (0.15)
Household size	2.85 (0.02)	2.84 (0.03)	2.88 (0.03)	2.94 (0.06)

*Note.* Results based on 50 multiply imputed data sets. Events includes participants from survey years 1993, 1998, 2002, 2012, and 2016. Activities includes participants from 1993, 1998, and 2002. Groups includes participants from 1993, 1994, 2004, and 2010. Interested non-attendees includes participants from 2012 and 2016. SE = standard error

In the logistic regression model, there was evidence for associations between several demographic factors and attending arts events (Table 2). Females had 24% higher odds (95% CI = 1.10–1.39) of attendance than males. In comparison to White participants, Black participants had 34% lower odds (95% CI = 0.55–0.78) of attendance. Participants who had never been married had 37% higher odds (95% CI = 1.14–1.63) of attendance than those who were married.

**Table 2:** Logistic regression models testing associations between demographic, socioeconomic, residential, and health exposures and the odds of arts engagement

	Model 1: Events n = 8684			Model 2: Activities n = 4372			Model 3: Groups n = 4268			Model 4: Interested non-attendes n = 2061		
	OR	95% CI	p	OR	95% CI	p	OR	95% CI	p	OR	95% CI	p
Age	1.01	0.98–1.03	0.629	1.01	0.98–1.03	0.605	**1.01**	**1.00–1.02**	**0.007**	1.00	0.99–1.01	0.850
Age (quadratic)	1.00	1.00–1.00	0.302	1.00	1.00–1.00	0.129	–	–	–	–	–	–
Female	**1.24**	**1.10–1.39**	**< 0.001**	**1.71**	**1.45–2.00**	**< 0.001**	**1.33**	**1.08–1.63**	**0.008**	1.19	0.90–1.58	0.215
Race/ethnicity												
White	1			1			1			1		
Black	**0.66**	**0.55–0.78**	**< 0.001**	**0.48**	**0.38–0.61**	**< 0.001**	0.94	0.66–1.33	0.718	0.92	0.61–1.39	0.696
Other	0.89	0.71–1.11	0.294	**0.70**	**0.51–0.96**	**0.028**	1.11	0.74–1.69	0.606	**0.56**	**0.35–0.89**	**0.015**
Marital status												
Married	1			1			1			1		
Separated	1.16	1.00–1.34	0.056	0.90	0.75–1.09	0.282	0.91	0.68–1.22	0.531	1.08	0.75–1.57	0.671
Never married	**1.37**	**1.14–1.63**	**0.001**	1.00	0.78–1.27	0.987	**1.58**	**1.18–2.11**	**0.002**	1.26	0.86–1.86	0.236
Employment status												
Employed	1			1			1			1		
Unemployed	0.93	0.73–1.19	0.561	**1.44**	**1.06–1.95**	**0.021**	0.79	0.50–1.23	0.286	1.41	0.85–2.34	0.179
Retired	1.15	0.93–1.43	0.200	1.10	0.83–1.45	0.523	1.26	0.85–1.85	0.249	0.89	0.57–1.39	0.605
Keeping house	0.82	0.67–1.01	0.057	1.13	0.87–1.46	0.350	1.38	0.95–2.00	0.089	0.95	0.62–1.47	0.831
Other	1.07	0.82–1.39	0.633	1.35	0.96–1.89	0.085	1.11	0.71–1.72	0.651	0.78	0.48–1.28	0.324
Family income												
$0–$9999	1			1			1			1		
$10,000–$19,999	**1.27**	**1.07–1.51**	**0.007**	0.84	0.65–1.07	0.162	1.15	0.77–1.74	0.492	1.13	0.76–1.68	0.536
$20,000–$29,999	**1.58**	**1.29–1.95**	**< 0.001**	0.96	0.72–1.27	0.756	1.54	0.97–2.45	0.067	1.11	0.66–1.87	0.698
$30,000–$49,999	**1.80**	**1.46–2.22**	**< 0.001**	0.87	0.65–1.17	0.358	1.42	0.91–2.23	0.122	1.14	0.73–1.78	0.557
$50,000+	**2.78**	**2.17–3.57**	**< 0.001**	0.81	0.58–1.13	0.211	1.42	0.89–2.26	0.137	1.03	0.49–2.15	0.940
Financial situation												
Not satisfied at all	1			1			–	–	–	1		
More or less satisfied	0.92	0.80–1.06	0.267	1.00	0.83–1.21	0.962	–	–	–	**0.70**	**0.52–0.96**	**0.028**
Pretty well satisfied	1.03	0.87–1.21	0.772	1.00	0.81–1.24	0.978	–	–	–	0.79	0.53–1.17	0.234
Social class												
Lower class	1			1			1			1		
Working class	1.20	0.94–1.53	0.145	1.20	0.86–1.69	0.285	1.21	0.64–2.30	0.558	1.02	0.65–1.62	0.916
Middle class	**1.52**	**1.16–1.97**	**0.002**	1.03	0.73–1.46	0.870	1.35	0.71–2.57	0.359	0.69	0.43–1.12	0.132
Upper class	1.52	0.99–2.35	0.058	0.92	0.56–1.50	0.743	1.67	0.77–3.62	0.195	0.29	0.08–1.09	0.066
Years of education	**1.19**	**1.16–1.22**	**< 0.001**	**1.08**	**1.05–1.12**	**< 0.001**	**1.15**	**1.10–1.20**	**< 0.001**	**1.11**	**1.05–1.17**	**< 0.001**
Parental years of education	**1.05**	**1.04–1.07**	**< 0.001**	**1.05**	**1.02–1.07**	**< 0.001**	**1.04**	**1.01–1.08**	**0.019**	1.03	0.99–1.08	0.147
General health rating												
Excellent	1			1			–	–	–	1		
Good	0.88	0.75–1.03	0.121	0.95	0.79–1.14	0.577	–	–	–	1.02	0.67–1.55	0.917
Fair	**0.70**	**0.58–0.85**	**< 0.001**	0.92	0.71–1.18	0.492	–	–	–	1.30	0.84–2.02	0.243
Poor	**0.48**	**0.34–0.67**	**< 0.001**	0.91	0.63–1.33	0.634	–	–	–	1.38	0.74–2.56	0.309
Level of urbanicity												
Med-large city	1			1			1			1		
Suburb	0.92	0.79–1.08	0.311	1.18	0.98–1.42	0.075	1.06	0.82–1.38	0.666	1.12	0.77–1.63	0.558
Unincorporated area	**0.79**	**0.64–0.96**	**0.020**	0.96	0.75–1.24	0.771	1.22	0.89–1.67	0.225	1.15	0.77–1.73	0.495
Small city or town	**0.69**	**0.58–0.83**	**< 0.001**	1.08	0.84–1.41	0.538	1.20	0.88–1.64	0.255	1.19	0.77–1.85	0.436
Smaller areas	**0.57**	**0.47–0.69**	**< 0.001**	0.98	0.75–1.28	0.874	0.96	0.63–1.45	0.836	0.63	0.39–1.02	0.061
Household size	**0.95**	**0.90–0.99**	**0.030**	1.02	0.95–1.09	0.572	0.99	0.91–1.08	0.811	0.96	0.87–1.05	0.350
Feels afraid in neighborhood	1.06	0.91–1.24	0.463	0.97	0.80–1.17	0.714	–	–	–	1.11	0.79–1.56	0.536
Survey year												
1	1			1			1			1		
2	**2.02**	**1.69–2.40**	**< 0.001**	0.89	0.74–1.06	0.194	0.73	0.52–1.03	0.074	1.04	0.79–1.38	0.757
3	**2.27**	**1.88–2.73**	**< 0.001**	1.05	0.88–1.26	0.568	**0.77**	**0.60–0.99**	**0.045**	–	–	–
4	**1.25**	**1.06–1.48**	**0.008**	–	–	–	**0.68**	**0.54–0.87**	**0.002**	–	–	–
5	1.05	0.87–1.26	0.624	–	–	–	–	–	–	–	–	–

*Note.* Survey year refers to different years for each arts outcome: for events 1 = 1993, 2 = 1998, 3 = 2002, 4 = 2012, 5 = 2016; for activities 1 = 1993, 2 = 1998, 3 = 2002; for groups 1 = 1993, 2 = 1994, 3 = 2004, 4 = 2010; and for interested non-attendees 1 = 2012, 2 = 2016. These numbers have been added for ease of presentation; years were used in analyses. For odds ratios, 1 indicates the reference category

There was evidence that several socioeconomic factors were associated with attendance. Compared to those with a family income of less than $10,000, participants in all other income groups had higher odds of attendance. The highest odds were in the highest income group. Subjective rating of social class was also associated with attendance, with higher classes associated with increasing odds. Each additional year of education was associated with 1.19 times higher odds (95% CI = 1.16–1.22) of attendance. Parental education was similarly associated with increased odds of attendance, although the estimated odds ratio was smaller (OR = 1.05, 95% CI = 1.04–1.07).

Two residential factors were associated with attendance. Compared to those living in medium to large cities, the odds of attendance reduced with decreasing level of urbanicity. The odds of attendance were lowest in smaller areas or open country. Additionally, for each additional person in the household, participants had 5% lower odds (95% CI = 0.90–0.99) of attendance. Participants who rated their health as fair (OR = 0.68, 95% CI = 0.56–0.83) or poor (OR = 0.47, 95% CI = 0.33–0.66) had lower odds of attending events than participants who rated their health as excellent.

Finally, the results suggested that event attendance varied across survey years, although there was no clear time trend. In comparison to 1993, the odds of attendance were higher in 1998, 2002, and 2012 but did not differ in 2016.

### Arts activities

Overall, 4372 individuals reported whether they had participated in arts activities. These individuals ranged in age from 18 to 89 years, with a mean age of 44.8 (SD = 17.0). About 53% were female and 81% were White (Table 1). On average, 54% reported participating in at least one arts activity in the last 12 months, and this was relatively stable across time (1993: 55%, 1998: 51%, 2002: 55%).

Fewer factors were associated with participation in arts activities than with attendance at arts events (Table 2). Females had 1.71 times higher odds (95% CI = 1.45–2.00) of participating than males. Both Black (OR = 0.48, 95% CI = 0.38–0.61) and individuals of Other races/ethnicities (OR = 0.70, 95% CI = 0.51–0.96) were less likely to report participating than those who were White. Individuals who were unemployed or not working had higher odds of participating than those working (OR = 1.44, 95% CI = 1.06–1.95). As with attending arts events, increased years of education (OR = 1.08, 95% CI = 1.05–1.12) and parental education (OR = 1.05, 95% CI = 1.02–1.07) were both associated with higher odds of participating in arts activities. There was no evidence that any other factors were associated with participation.

### Creative groups

Membership of creative groups was reported by 4268 participants, who were similar demographically to participants who reported other arts outcomes (Table 1). Membership in creative groups was lower than attendance at events or participation in activities. Overall, 19% of participants reported being a member of a creative group, and this may have decreased over time (1993: 20%, 1994: 16%, 2004: 18%, 2010: 17%).

Despite a lower proportion of participants being members of creative groups, membership was associated with similar factors to arts activities (Table 2). Females had 1.33 times higher odds (95% CI = 1.08–1.63) of membership than males. There was also evidence that the odds of membership increased with more education (OR = 1.15, 95% CI = 1.10–1.20) and parental education (OR = 1.04, 95% CI = 1.01–1.08). In contrast to arts activities, those who were never married had 1.58 times higher odds (95% CI = 1.18–2.11) of membership than married participants and the odds of membership increased with age (OR = 1.01, 95% CI = 1.00–1.02). Finally, there was evidence that membership decreased over time, with the odds decreasing by 32% (95% CI = 0.54–0.87) from 1993 to 2010.

### Interested non-attendees

Overall, 2061 participants reported whether there was an arts event that they had wanted to go to but did not attend, 29% of whom were interested non-attendees. The proportion of interested non-attendees remained consistent across years (2012: 29%, 2016: 30%).

As with attendance at arts events, there was evidence that being an interested non-attendee was associated with race/ethnicity and years of education (Table 2). Other races/ethnicities had lower odds of being an interested non-attendee than White individuals (OR = 0.56, 95% CI = 0.35–0.89), and the odds of interested non-attendance increased with level of education (OR = 1.11, 95% CI = 1.05–1.17). However, in contrast to event attendance, those who were more or less satisfied with their financial situation had lower odds of being an interested non-attendee than those who were not satisfied at all (OR = 0.70, 95% CI = 0.52–0.96). There was no evidence that being an interested non-attendee was associated with gender, marital status, employment status, family income, social class, parental education, level of urbanicity, household size, or general health rating.

### Change across survey years

Next, we tested whether associations between arts engagement outcomes and age, sex, race/ethnicity, class, and income differed over time. There was no evidence for interactions between survey year and any exposures on participation in arts activities, membership of creative groups, or being an interested non-attendee (Supplementary Table 5). There was also no evidence for interactions between survey year and age, class, or income on arts event attendance.

**Fig. 1:**
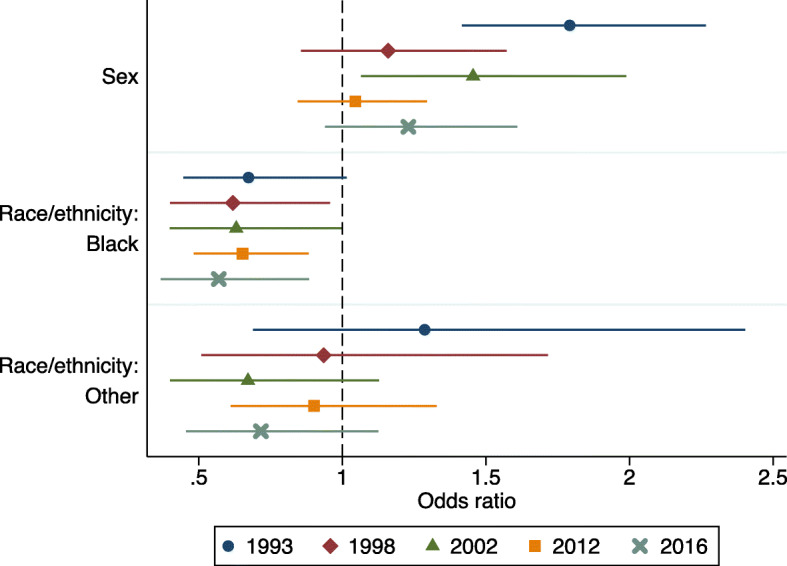
Results of subgroup analyses, with logistic regression models testing associations between exposures and the odds of attending arts events separately in each survey year (1993 *n* = 1590, 1998 *n* = 1432, 2002 *n* = 1355, 2012 *n* = 2838, 2016 *n* = 1469). Odds ratios and 95% confidence intervals are displayed. For associations between sex and arts events, the odds ratio represents attendance in females compared to males. For associations between race/ethnicity and arts events, White is the reference category. Associations were estimated in the full logistic regression models (including all exposures as shown in Table 2), but only results for sex and race/ethnicity are presented

However, there was evidence for an interaction between survey year and sex on event attendance. There was no linear time trend, as females had higher odds of attendance than males in 1993 and 2002 but there were no sex differences in other survey years (Fig. 1; Supplementary Table 6). There was also evidence for an interaction between survey year and race/ethnicity on event attendance. Black participants had lower odds of attending than White participants, and this difference increased over time (Fig. 1; Supplementary Table 6).

### Sensitivity analyses

We have reported findings based on imputed data but the results of analyses did not vary when limited to complete cases, as shown in Supplementary Table 4. In our sensitivity analysis, limiting the sample to the most homogenous definitions of arts event attendance (i.e. excluding participants from 1993) did not substantially alter our findings (Supplementary Table 7).

## Discussion

In this study, we examined whether there are social inequalities in engagement in the arts, as found in other health-related behaviors [3–5]. Between 1993 and 2016, approximately half of our sample reported attending arts events, and a similar proportion participated in arts activities. In the smaller sample of individuals who completed the GSS in 2012 and 2016, another one third were interested non-attendees, who had been interested in attending an event in the last year but had not gone to it. Fewer people were members of creative groups, with approximately one fifth of the sample between 1993 and 2010 reporting group membership. Several demographic factors were consistently associated with engagement in the arts. For example, engagement was higher in females than males, and married individuals were less likely to engage than those who had never married. Attendance at arts events and participation in arts activities also differed according to race/ethnicity, although creative group membership did not. Socioeconomic factors showed mixed associations with the different types of arts engagement. Higher levels of education and parental education were consistently associated with all types of engagement. Attendance at arts events was also associated with higher income and social class, better health, and living in more urban areas. However, being an interested non-attendee of arts events was not associated with these factors. In contrast to arts events, we found no evidence that income, social class, health, or urbanicity were associated with participation in arts activities and groups. Most of our findings are consistent with previous research demonstrating that a number of demographic and socioeconomic factors are associated with engagement in the arts [13]. Our findings further advance previous research by using a broader definition of arts to more accurately reflect the breadth of engagement in the US.

The associations between several demographic factors, such as sex and marital status, and engagement in all forms of the arts are consistent with previous evidence [19, 40–44]. We also found that race/ethnicity was more strongly associated with participation in arts activities than events, as shown previously [22]. This association was independent of socioeconomic factors, so is unlikely to be explained by over-representation of ethnic minorities in lower socioeconomic status groups [45]. A report that also used GSS data found that lower attendance at arts events by racial/ethnic minorities may be a result of barriers such as being unable to get to the venue and not having anyone to go with [23]. These individuals were also more likely to state celebrating their cultural heritage as a reason for attending events than those who were White [23, 46]. However, in this study, we found that Other races/ethnicities were also less likely to be interested non-attendees of arts events than White individuals. Although this could be a result of the way in which arts events were defined (limited to music, theatre, or dance performances or art exhibits), it may also indicate that some ethnic/racial groups are less interested in attending arts events. A lack of cultural equity, cultural relevance, interest, and inequalities in access are therefore likely to contribute to the racial/ethnic differences in arts engagement.

Overall, our findings support previous evidence that education is most strongly associated with engagement in the arts [12, 13, 18–24]. However, contrary to some recent evidence, we did not find that education was more strongly associated with attending events than other forms of arts engagement [25]. Education may increase engagement by helping to cultivate cultural tastes and preferences, raising awareness of activities, and increasing cognitive capacity to engage [47]. Arts education specifically may also contribute to this association, as it is strongly related to both level of education and arts engagement [20, 27, 32, 48, 49]. We found a similar association with parental education, independent of the individual’s own education, although the magnitude of association was smaller. This indicates that childhood socioeconomic status continues to influence engagement in the arts throughout the lifecourse. Children of parents with more education may benefit from increased access to the arts during development and may be more likely to receive arts education in childhood (e.g. learning to play an instrument [30]). These individuals may therefore have more training and experience, enabling them to participate in more highly skilled arts activities (e.g. orchestras).

Consistent with previous evidence for a social gradient in arts engagement, we found that attendance at arts events was less likely with lower income and social class, poorer health, and less urban areas. As being an interested non-attendee was not associated with these factors, they are likely to be barriers specifically to attendance. Individuals across the range of incomes, social classes, health, and levels of urbanicity were interested in attending events at similar rates, but actual attendance differed according to these factors. Previously, individuals with lower household income and social class were more likely to report barriers to attending events of cost and difficulty of getting to a venue, as well as a lack of time [23, 46]. Other research has demonstrated that individuals with poorer physical health may experience more barriers affecting their perceived capabilities to engage [30]. Areas that are more urban, such as cities, are likely to have a larger range of arts events on offer, including at a variety of times and costs as well as appealing to a broader audience, and events may be more geographically dispersed or easier to attend using public transport. Urbanicity can thus be interpreted as a proxy measure for the availability of arts events. However, there are also likely to be area-level factors related to the availability and accessibility of the arts that, although not measured in the GSS, require further investigation. In contrast to arts events, we found no evidence that income, social class, health, or urbanicity were associated with participation in arts activities and groups. These types of engagement may be more widely available, include more diverse activities, be cheaper to participate in, and often do not require attendance at a specific venue, which may be hard to reach or not generally attended by certain groups.

There was some mixed evidence for a social gradient in interest in arts events. Individuals with higher levels of education were more likely to be interested non-attendees, as were people who were more or less satisfied with their financial situation (compared to those who were not satisfied at all). Previous research has suggested that of the different types of arts engagement, education is most strongly associated with highbrow cultural events [25], which could explain the association with interest in events. It is unclear why we found evidence for an association with financial satisfaction. We might conclude that individuals who were satisfied with their financial situation were not interested non-attendees because they were financially able to attend any events of interest, but we found no evidence that financial satisfaction was associated with actual event attendance. Additionally, there was no evidence that being an interested non-attendee was associated with income or differed between those who were pretty well satisfied and not at all satisfied with their financial situation. The relationship between interest in the arts, subjective measures of satisfaction with financial situation, and more objective measures of income thus requires further investigation.

We also investigated changing patterns of arts engagement as there has been concern that arts participation is decreasing in the US [11, 22, 32]. We found some evidence that event attendance changed over time, but this was likely a result of changes in the measure of event attendance, as there was no linear trend. In contrast, group membership decreased over time. Additionally, the racial disparity in event attendance, with an over-representation of White individuals compared to those of racial/ethnic minorities, increased from 1993 to 2016. These increasing racial/ethnic inequalities in arts event attendance were independent of other socioeconomic factors such as income and education. However, given the nature of structural racism, this finding should be interpreted cautiously and requires replication in studies with consistent measures of event attendance. As this study spanned a period of 23 years, with event attendance and group membership measured at different times, specific social and economic events in each year could also have contributed to the changing patterns of arts engagement.

Our findings have implications for understanding health and social inequalities in the US. A number of the factors that we have identified as associated with arts engagement are also associated with inequalities in access to health care and health outcomes [14–17]. This could be because arts engagement is a correlate of health, with both representing a form of capital that can be obtained by individuals with more material resources, such as income, and non-material resources, such as social support [47]. Consistent with this, we found evidence that poorer self-reported health was associated with lower attendance at arts events, although it was not associated with interest in attending events or participation in arts activities. Arts engagement could also represent a health behaviour that leads to improved health outcomes. There is growing evidence that engagement with the arts can lead to a range of health benefits, independent of demographic and socioeconomic factors [9, 50]. It is thus concerning that we have found evidence for differential engagement in the arts. Future research should explore why engagement is lower in these groups, in particular males, racial/ethnic minorities, and those with lower education. This is particularly important given that previous efforts to reduce inequalities in access to cultural events by expanding facilities and offering free tickets in Brazil have not been successful [51]. Future research could also investigate whether removing other barriers to engagement, such as providing the arts online to avoid high prices and reduce time constraints, could increase levels of engagement [52]. This could then support the development of interventions to promote engagement in the arts, and test whether this leads to improvements in health outcomes.

This study has a number of strengths. The GSS was a large nationally representative sample and we included several measures of arts engagement. Although the GSS has previously been used to study arts engagement [23, 43], research has not generally examined membership of creative groups in comparison to other forms of engagement or combined data across as many waves of the GSS as in this study. We tested a range of factors that may be associated with arts engagement, and mutually adjusted for these variables in our models. Using multiple imputation means that missing data should not have influenced our findings. However, this study also has a number of limitations. We tested cross-sectional associations and thus cannot rule out the possibility of inverse causality. There are some factors, such as health, which may have a bidirectional association with arts engagement. Additionally, the GSS did not measure attendance at arts events consistently across waves, which is likely to explain the association we found between event attendance and survey year. A broader definition of arts events was used in later years. However, when limiting our analyses just to this broader definition, our findings were consistent. Although our measures of arts engagement were more inclusive than in many previous studies, they were likely still too narrow. Standard arts engagement questions are not able to capture arts engagement in some immigrant communities [35], and also typically do not cover engagement in digital or electronic arts activities such as graphic design, photography, film-making, and music production. This could have contributed to our findings of lower arts engagement in individuals who were not White and under-represented arts engagement amongst younger generations. Future research should aim to measure diverse aspects of arts engagement, particularly as the US moves towards a majority-minority society, in which the non-Hispanic white population will no longer form the majority of the US population [53].

## Conclusions

Given the potential importance of engagement in the arts for health and wellbeing [9], individuals should be provided with equal opportunities to participate. Our findings indicate that social determinants may influence engagement in the arts throughout the life course. Encouraging arts activities and creative group membership may provide one way of widening participation and reducing social inequalities in arts engagement. It will also be important to recognize that lack of participation may not merely be due to a lack of interest or motivation but may be influenced by structural barriers, such as racism, or a lack of opportunities. Indeed, the nature of many arts activities that take place in well defined arts spaces are rooted in white supremacy, creating a foundational barrier for Black, Indigeouns and other people of color (BIPOC) groups. Future research is needed to identify what these barriers are and how they can be removed. This is particularly important in the wake of COVID-19, given the closure of many arts venues and the disproportionate effect on BIPOC individuals and those of lower socioeconomic status [28, 54–56].

## Supplementary Information

**Additional file 1.**

## Data Availability

The dataset supporting the conclusions of this article is available in the GSS repository, https://gss.norc.org/get-the-data/stata.
