# Supplementary material for: Who engages in the arts in the United States? A comparison of several types of engagement using data from The General Social Survey
Source: BMC Public Health. 2021 Jul 8;21:1349. doi: 10.1186/s12889-021-11263-0 (PMC8264486; doi:10.1186/s12889-021-11263-0)
Supplement: Supplementary file 1 — Additional file 1. [file 12889_2021_11263_MOESM1_ESM.docx]

**Supplementary Materials**

**Sample selection**

Participants were drawn from the General Social Survey (GSS) waves at which arts outcomes were measured, specifically 1993, 1994, 1998, 2002, 2004, 2010, 2012 and 2016. Each of these waves included a sample of unique individuals.

The GSS includes a repeated cross-sectional study with waves measuring arts outcomes in 1993, 1994, 1998, 2002, 2004 and 2016. Each survey year was an independently drawn sample of English-speaking individuals living in non-institutional arrangements. From 2006 onwards, Spanish-speakers were added to the target population. Full probability sampling was employed, and surveys sub-sampled non-respondents from 2004 onwards.

However, the GSS also includes a rotating panel study, which measured arts outcomes in 2010 and 2012. The panel element of the GSS included three rolling panels following up participants across three waves, each two years apart. Each panel began with an independently drawn sample of English- and Spanish-speaking persons living in non-institutional arrangements or households within the United States. Respondents were drawn from full probability samples and all three years of panel initialization featured sub-sampling of non-respondents. Participants from the panel study only completed arts outcome measures at one time-point and were therefore treated as cross-sectional samples. The 2010 panel sample were in their third and final wave, having been recruited in 2006. The 2012 panel sample included participants in their second wave (recruited in 2010) as well as participants in their third wave (recruited in 2008).

Supplementary Table 1 outlines the total GSS sample size in the waves in which arts engagement were measured. Of the total GSS sample, varying proportions of participants completed the different arts engagement outcomes. A split-sample design was introduced from 1994, meaning approximately half of participants completed questions on arts engagement in each wave. Attendance at arts events was measured in 1993, 1998, 2002, 2012, and 2016. Across these waves, GSS included a total of 14,890 participants, 8684 (58%) of whom answered questions on attendance at arts events. Engagement in arts activities was measured in 1993, 1998, and 2002 and, in these years, the same subgroups of participants completed events and activities measures. Across 1993, 1998, and 2002, 7203 individuals participated in the GSS, 4372 (61%) of whom were asked about engagement in arts activities. Membership of creative groups was measured in 1993, 1994, 2004, and 2010. In total, there were 12,311 participants across these waves, 4268 (35%) of whom answered questions on creative group membership. Finally, a subsample of participants in 2012 and 2016 who were asked about attendance at arts events were also asked whether they were interested non-attendees. In total, there were 7,687 participants across these waves, 2,061 (27%) of whom did not attend an arts event and answered questions on being an interested non-attendee.

**Supplementary Table 1** *Sample selection procedure.*

| **GSS wave** | **Total N** | **N completed each arts engagement measure** | | | |
| --- | --- | --- | --- | --- | --- |
|  |  | **Events** | **Activities** | **Groups** | **Interested**  **non-attendees** |
| **1993** | 1606 | 1590 | 1586 | 1051 | 0 |
| **1994** | 2992 | 0 | 0 | 508 | 0 |
| **1998** | 2832 | 1432 | 1428 | 0 | 0 |
| **2002** | 2765 | 1355 | 1358 | 0 | 0 |
| **2004** | 2812 | 0 | 0 | 1466 | 0 |
| **2010** | 4901 | 0 | 0 | 1243 | 0 |
| **2012** | 4820 | 2838 | 0 | 0 | 1332 |
| **2016** | 2867 | 1469 | 0 | 0 | 729 |
| **Total** | **25595** | **8684** | **4372** | **4268** | **2061** |

**Supplementary Table 2** *Full details of the questions used to measure arts and cultural engagement outcomes.*

| **Wave** | **Questions** |
| --- | --- |
| **Events** | |
| 1993 | Next I'd like to ask about some leisure or recreational activities that people do during their free time. As I read each activity, can you tell me if it is something you have done in the past twelve months?   - Visit an art museum or gallery. - Go to a live ballet or dance performance, not including school performances. - Go to a classical music or opera performance, not including school performances. |
| 1998  2002 | Next I'd like to ask about some leisure or recreational activities that people do during their free time. As I read each activity, can you tell me if it is something you have done in the past twelve months?   - Visit an art museum or gallery. - Go to a live ballet or dance performance, not including school performances. - Go to a classical music or opera performance, not including school performances. - Go to a live performance of popular music like rock, country, or rap, not including school performances. - Go to a live performance of a non-musical stage play, not including school performances. |
| 2012  2016 | - With the exception of elementary or high school performances, did you go to a live music, theater, or dance performance, during the last 12 months? - During the last 12 months, did you go to an art exhibit, such as paintings, sculpture, textiles, graphic design, or photography? |
| A**ctivities** | |
| 1993  1998  2002 | Next I'd like to ask about some leisure or recreational activities that people do during their free time. As I read each activity, can you tell me if it is something you have done in the past twelve months?   - Make art or craft objects such as pottery, woodworking, quilts, or paintings. - Take part in a music, dance, or theatrical performance. - Play a musical instrument like a piano, guitar, or violin. |
| **Groups** | |
| 1993  1994  2004  2010 | We would like to know something about the groups and organizations to which individuals belong. Here is a list of various kinds of organizations. Could you tell me whether or not you are a member of each type?   - Hobby or garden clubs - Literary, art, discussion, or study groups |
| **Interested non-attendance** | |
| 2012  2016 | - During the last 12 months, was there a performance or exhibit that you wanted to go to but did not? |

*Note.* Response options for all questions in all waves were: Yes, No, Don’t know.

**Supplementary Table 3** *Proportion of missing data in each exposure in the four analytical samples.*

|  | **Events**  n=8684 | **Activities**  n=4372 | **Groups**  n=4268 | **Interested**  **non-attendees**  n=2061 |
| --- | --- | --- | --- | --- |
| Age | 0.7% | 0.3% | 0.6% | 1.7% |
| Female | 0 | 0 | 0 | 0 |
| Race | 0 | 0 | 0 | 0 |
| Marital status | 0.03% | 0.02% | 0.02% | 0.05% |
| Work status | 0.01% | 0 | 0.05% | 0.05% |
| Family income | 9.3% | 10.1% | 10.0% | 10.1% |
| Satisfaction with financial situation | 0.2% | 0.3% | - | 0.2% |
| Social class | 0.7% | 0.7% | 0.6% | 0.9% |
| Years of education | 0.2% | 0.3% | 0.1% | 0.2% |
| Parental years of education | 6.8% | 9.3% | 8.4% | 6.0% |
| General health rating | 28.5% | 22.3% | - | 34.2% |
| Level of urbanicity | 0 | 0 | 0 | 0 |
| Household size | 0 | 0 | 0 | 0 |
| Feels afraid in neighborhood | 34.3% | 33.4% | - | 34.9% |

**Supplementary Table 4** *Logistic regression models testing associations between demographic, socioeconomic, residential, and health exposures and the odds of three types of arts engagement as well as being an interested non-attendee, in complete cases only.*

|  | **Model 1: Events**  n=4825 | | | **Model 2: Activities**  n=2387 | | | **Model 3: Groups**  n=3523 | | | **Model 4: Interested  non-attendees** n=1123 | | |
| --- | --- | --- | --- | --- | --- | --- | --- | --- | --- | --- | --- | --- |
|  | **OR** | **95% CI** | **p** | **OR** | **95% CI** | **p** | **OR** | **95% CI** | **p** | **OR** | **95% CI** | **p** |
| Age | 1.01 | 0.97-1.04 | 0.642 | 1.00 | 0.96-1.04 | 0.926 | **1.01** | **1.00-1.02** | **0.005** | 1.01 | 0.99-1.02 | 0.503 |
| Age (quadratic) | 1.00 | 1.00-1.00 | 0.425 | 1.00 | 1.00-1.00 | 0.539 | - | - | - | - | - | - |
| Female | **1.36** | **1.16-1.60** | **<0.001** | **1.86** | **1.49-2.32** | **<0.001** | **1.28** | **1.02-1.60** | **0.030** | 1.20 | 0.84-1.72 | 0.322 |
| Race |  |  |  |  |  |  |  |  |  |  |  |  |
| White | 1 |  |  | 1 |  |  | 1 |  |  | 1 |  |  |
| Black | **0.71** | **0.56-0.91** | **0.006** | **0.50** | **0.37-0.68** | **<0.001** | 0.93 | 0.62-1.38 | 0.702 | 0.92 | 0.51-1.69 | 0.798 |
| Other | 0.90 | 0.66-1.23 | 0.515 | 0.77 | 0.48-1.24 | 0.284 | 1.29 | 0.81-2.05 | 0.284 | 0.54 | 0.30-0.97 | 0.039 |
| Marital status |  |  |  |  |  |  |  |  |  |  |  |  |
| Married | 1 |  |  | 1 |  |  | 1 |  |  | 1 |  |  |
| Separated/divorced/widowed | **1.25** | **1.01-1.56** | **0.041** | **0.75** | **0.57-0.98** | **0.033** | 1.04 | 0.75-1.43 | 0.829 | 1.32 | 0.78-2.26 | 0.300 |
| Never married | **1.44** | **1.13-1.83** | **0.003** | 0.92 | 0.68-1.26 | 0.610 | **1.75** | **1.26-2.44** | **0.001** | 1.50 | 0.89-2.54 | 0.128 |
| Work status |  |  |  |  |  |  |  |  |  |  |  |  |
| Employed | 1 |  |  | 1 |  |  | 1 |  |  | 1 |  |  |
| Unemployed/not working | 1.01 | 0.71-1.44 | 0.956 | **1.88** | **1.20-2.95** | **0.006** | 0.77 | 0.48-1.26 | 0.300 | **1.94** | **1.02-3.67** | **0.042** |
| Retired | 1.05 | 0.77-1.43 | 0.753 | 1.24 | 0.85-1.81 | 0.260 | 1.19 | 0.78-1.81 | 0.411 | 0.65 | 0.36-1.14 | 0.133 |
| Keeping house | 0.78 | 0.58-1.05 | 0.103 | 1.29 | 0.90-1.83 | 0.164 | 1.50 | 0.98-2.30 | 0.063 | 1.10 | 0.62-1.95 | 0.734 |
| Other | 0.90 | 0.61-1.32 | 0.587 | 1.17 | 0.72-1.90 | 0.519 | 1.31 | 0.78-2.18 | 0.304 | 0.78 | 0.36-1.68 | 0.524 |
| Family income |  |  |  |  |  |  |  |  |  |  |  |  |
| $0-$9,999 | 1 |  |  | 1 |  |  | 1 |  |  | 1 |  |  |
| $10,000-$19,999 | 1.17 | 0.91-1.51 | 0.218 | 1.04 | 0.75-1.45 | 0.808 | 1.20 | 0.75-1.92 | 0.444 | 1.22 | 0.71-2.08 | 0.464 |
| $20,000-$29,999 | **1.51** | **1.12-2.03** | **0.007** | 1.09 | 0.73-1.62 | 0.685 | **1.78** | **1.04-3.05** | **0.036** | 0.82 | 0.43-1.60 | 0.565 |
| $30,000-$49,999 | **1.96** | **1.42-2.70** | **<0.001** | 1.14 | 0.77-1.69 | 0.507 | 1.64 | 0.99-2.74 | 0.056 | 1.22 | 0.66-2.27 | 0.528 |
| $50,000+ | **2.67** | **1.89-3.77** | **<0.001** | 1.01 | 0.64-1.60 | 0.963 | 1.65 | 0.97-2.80 | 0.066 | 0.80 | 0.27-2.34 | 0.679 |
| Satisfaction with financial situation |  |  |  |  |  |  |  |  |  |  |  |  |
| Pretty well satisfied | 1 |  |  | 1 |  |  | - | - | - | 1 |  |  |
| More or less satisfied | 0.95 | 0.78-1.16 | 0.645 | 1.01 | 0.78-1.31 | 0.952 | - | - | - | 0.72 | 0.47-1.11 | 0.136 |
| Not satisfied at all | 1.01 | 0.79-1.29 | 0.930 | 0.89 | 0.68-1.16 | 0.397 | - | - | - | 0.97 | 0.58-1.62 | 0.901 |
| Social class |  |  |  |  |  |  |  |  |  |  |  |  |
| Lower class | 1 |  |  | 1 |  |  | 1 |  |  | 1 |  |  |
| Working class | 1.19 | 0.83-1.70 | 0.344 | 1.53 | 0.93-2.50 | 0.091 | 1.19 | 0.57-2.47 | 0.642 | 1.19 | 0.68-2.09 | 0.542 |
| Middle class | **1.63** | **1.11-2.39** | **0.013** | 1.22 | 0.74-2.03 | 0.437 | 1.21 | 0.58-2.51 | 0.609 | 0.74 | 0.42-1.33 | 0.316 |
| Upper class | **2.25** | **1.15-4.40** | **0.017** | 0.86 | 0.42-1.78 | 0.691 | 1.31 | 0.55-3.09 | 0.537 | 0.10 | 0.01-1.40 | 0.087 |
| Years of education | **1.20** | **1.15-1.24** | **<0.001** | **1.08** | **1.04-1.13** | **<0.001** | **1.14** | **1.08-1.20** | **<0.001** | **1.10** | **1.01-1.20** | **0.039** |
| Parental years of education | **1.04** | **1.02-1.07** | **0.002** | **1.06** | **1.02-1.09** | **0.001** | **1.05** | **1.01-1.09** | **0.006** | 1.03 | 0.98-1.09 | 0.246 |
| General health rating |  |  |  |  |  |  |  |  |  |  |  |  |
| Excellent | 1 |  |  | 1 |  |  | - | - | - | 1 |  |  |
| Good | 1.00 | 0.83-1.21 | 0.990 | 1.01 | 0.81-1.25 | 0.961 | - | - | - | 1.02 | 0.62-1.68 | 0.943 |
| Fair | **0.78** | **0.61-0.98** | **0.035** | 0.93 | 0.69-1.26 | 0.643 | - | - | - | 1.18 | 0.71-1.98 | 0.520 |
| Poor | **0.41** | **0.26-0.64** | **<0.001** | 1.30 | 0.82-2.06 | 0.267 | - | - | - | 1.36 | 0.64-2.92 | 0.423 |
| Level of urbanicity |  |  |  |  |  |  |  |  |  |  |  |  |
| Med-large city (50,000+) | 1 |  |  | 1 |  |  | 1 |  |  | 1 |  |  |
| Suburb | 0.81 | 0.66-1.01 | 0.063 | 1.03 | 0.83-1.29 | 0.767 | 1.06 | 0.79-1.42 | 0.695 | 1.22 | 0.72-2.05 | 0.456 |
| Unincorporated area | **0.69** | **0.51-0.92** | **0.013** | 0.98 | 0.70-1.37 | 0.908 | 1.22 | 0.85-1.76 | 0.281 | 1.22 | 0.67-2.21 | 0.514 |
| Small city or town | **0.66** | **0.51-0.87** | **0.003** | 1.18 | 0.86-1.63 | 0.301 | 1.22 | 0.85-1.75 | 0.275 | 1.53 | 0.81-2.87 | 0.188 |
| Smaller areas or country | **0.57** | **0.43-0.76** | **<0.001** | 1.05 | 0.72-1.54 | 0.780 | 0.93 | 0.56-1.54 | 0.764 | 0.55 | 0.29-1.07 | 0.078 |
| Household size | 0.97 | 0.91-1.05 | 0.454 | 1.03 | 0.94-1.13 | 0.489 | 0.97 | 0.88-1.07 | 0.555 | 0.99 | 0.88-1.12 | 0.902 |
| Feels afraid in neighborhood | 1.03 | 0.86-1.24 | 0.734 | 1.08 | 0.87-1.34 | 0.503 | - | - | - | 1.17 | 0.78-1.75 | 0.443 |
| Survey year |  |  |  |  |  |  |  |  |  |  |  |  |
| 1 | 1 |  |  | 1 |  |  | 1 |  |  | 1 |  |  |
| 2 | **2.06** | **1.60-2.64** | **<0.001** | 0.93 | 0.74-1.18 | 0.563 | 0.78 | 0.53-1.15 | 0.207 | 1.05 | 0.68-1.60 | 0.833 |
| 3 | **2.47** | **1.90-3.21** | **<0.001** | 1.09 | 0.88-1.35 | 0.430 | 0.78 | 0.59-1.02 | 0.073 | - | - | - |
| 4 | 1.15 | 0.91-1.45 | 0.254 | - | - | - | **0.63** | **0.48-0.83** | **0.001** | - | - | - |
| 5 | 1.10 | 0.84-1.44 | 0.476 | - | - | - | - | - | - | - | - | - |

*Note.* Survey year refers to different years for each arts outcome: for events 1=1993, 2=1998, 3=2002, 4=2012, 5=2016; for activities 1=1993, 2=1998, 3=2002; for groups 1=1993, 2=1994, 3=2004, 4=2010; and for interested non-attendees 1=2012, 2=2016. These numbers have been added for ease of presentation; years were used in analyses. For odds ratios, 1 indicates the reference category.

**Supplementary Table 5** *Logistic regression models testing whether there was evidence that associations between arts engagement outcomes and age, sex, race/ethnicity, class, and income differed over time (using imputed data).*

|  | **Events** | | | **Activities** | | | **Groups** | | | **Interested  non-attendees** | | |
| --- | --- | --- | --- | --- | --- | --- | --- | --- | --- | --- | --- | --- |
| **Interaction with survey year** | **OR** | **95% CI** | **p** | **OR** | **95% CI** | **p** | **OR** | **95% CI** | **p** | **OR** | **95% CI** | **p** |
| Age | 1.00 | 1.00-1.00 | 0.204 | 1.00 | 1.00-1.00 | 0.871 | 1.00 | 1.00-1.00 | 0.076 | 1.00 | 1.00-1.00 | 0.950 |
| Sex | **0.99** | **0.98-1.00** | **0.038** | 1.00 | 0.96-1.04 | 0.973 | 1.00 | 0.98-1.03 | 0.797 | 0.92 | 0.82-1.04 | 0.199 |
| Race/ethnicity: Black | **0.98** | **0.96-1.00** | **0.034** | 1.00 | 0.94-1.06 | 0.949 | 1.01 | 0.96-1.06 | 0.628 | 0.99 | 0.82-1.20 | 0.937 |
| Race/ethnicity: Other | **0.97** | **0.94-1.00** | **0.024** | 1.01 | 0.92-1.10 | 0.879 | **0.93** | **0.88-0.99** | **0.017** | 0.83 | 0.65-1.05 | 0.111 |
| Class | 1.00 | 0.99-1.02 | 0.381 | 0.99 | 0.96-1.01 | 0.336 | 1.00 | 0.98-1.02 | 0.846 | 0.96 | 0.87-1.06 | 0.423 |
| Income | 1.00 | 1.00-1.01 | 0.469 | 1.00 | 0.99-1.01 | 0.969 | 1.00 | 0.99-1.01 | 0.348 | 0.98 | 0.92-1.04 | 0.437 |

*Note.* Interaction terms that include survey year and each sociodemographic factor as continuous variables (except race) are reported to indicate whether there is evidence for an interaction. Survey years were: 1993, 1998, 2002, 2012, and 2016 for events; 1993, 1998, and 2002 for activities; 1993, 1994, 2004, and 2010 for groups; and 2012 and 2016 for interested non-attendees.

**Supplementary Table 6** *Results of subgroup analyses, with logistic regression models testing associations between sex and race/ethnicity and the odds of attending arts events separately in each survey year (using imputed data).*

| **Survey year** | **Sex: Female** | | | **Race/ethnicity: Black** | | | **Race/ethnicity: Other** | | |
| --- | --- | --- | --- | --- | --- | --- | --- | --- | --- |
|  | **OR** | **95% CI** | **p** | **OR** | **95% CI** | **p** | **OR** | **95% CI** | **p** |
| 1993 | **1.79** | **1.42-2.27** | **<0.001** | 0.67 | 0.45-1.02 | 0.059 | 1.29 | 0.69-2.40 | 0.425 |
| 1998 | 1.16 | 0.86-1.57 | 0.335 | **0.62** | **0.40-0.96** | **0.031** | 0.93 | 0.51-1.72 | 0.826 |
| 2002 | **1.46** | **1.07-1.99** | **0.019** | **0.63** | **0.40-1.00** | **0.048** | 0.67 | 0.40-1.13 | 0.130 |
| 2012 | 1.05 | 0.84-1.29 | 0.684 | **0.65** | **0.48-0.88** | **0.006** | 0.90 | 0.61-1.33 | 0.598 |
| 2016 | 1.23 | 0.94-1.61 | 0.129 | **0.57** | **0.37-0.88** | **0.013** | 0.72 | 0.46-1.13 | 0.145 |

*Note.* This table presents the findings shown in Figure 1 in the main text. Arts event attendance was the outcome in all models. For sex, the reference category was male. For race/ethnicity, the reference category was White. 1993 n=1590, 1998 n=1432, 2002 n=1355, 2012 n=2838, 2016 n=1469.

**Supplementary Table 7** *Logistic regression model testing associations between demographic, socioeconomic, residential, and health exposures and the odds of attending an arts event, limited only to the broad definition of arts events measured from 1998 to 2016 (using imputed data).*

|  | **OR** | **95% CI** | **p** |
| --- | --- | --- | --- |
| Age | 1.00 | 0.98-1.03 | 0.968 |
| Age (quadratic) | 1.00 | 1.00-1.00 | 0.539 |
| Female | **1.15** | **1.01-1.31** | **0.035** |
| Race |  |  |  |
| White | 1 |  |  |
| Black | **0.64** | **0.53-0.78** | **<0.001** |
| Other | 0.84 | 0.66-1.07 | 0.163 |
| Marital status |  |  |  |
| Married | 1 |  |  |
| Separated | 1.16 | 0.98-1.37 | 0.078 |
| Never married | **1.36** | **1.12-1.64** | **0.002** |
| Work status |  |  |  |
| Employed | 1 |  |  |
| Unemployed | 0.99 | 0.75-1.31 | 0.935 |
| Retired | 1.21 | 0.96-1.53 | 0.109 |
| Keeping house | **0.75** | **0.60-0.95** | **0.017** |
| Other | 1.11 | 0.83-1.49 | 0.470 |
| Family income |  |  |  |
| $0-$9,999 | 1 |  |  |
| $10,000-$19,999 | **1.36** | **1.12-1.66** | **0.002** |
| $20,000-$29,999 | **1.63** | **1.29-2.05** | **<0.001** |
| $30,000-$49,999 | **1.87** | **1.48-2.36** | **<0.001** |
| $50,000+ | **2.83** | **2.14-3.74** | **<0.001** |
| Satisfaction with financial situation |  |  |  |
| Not satisfied at all | 1 |  |  |
| More or less satisfied | 0.99 | 0.84-1.16 | 0.864 |
| Pretty well satisfied | 1.05 | 0.88-1.27 | 0.574 |
| Social class |  |  |  |
| Lower class | 1 |  |  |
| Working class | 1.09 | 0.84-1.42 | 0.519 |
| Middle class | **1.40** | **1.05-1.88** | **0.023** |
| Upper class | 1.29 | 0.81-2.06 | 0.282 |
| Years of education | **1.18** | **1.14-1.21** | **<0.001** |
| Parental years of education | **1.06** | **1.04-1.08** | **<0.001** |
| General health rating |  |  |  |
| Excellent | 1 |  |  |
| Good | 0.90 | 0.75-1.09 | 0.287 |
| Fair | **0.69** | **0.55-0.86** | **0.001** |
| Poor | **0.47** | **0.32-0.68** | **<0.001** |
| Level of urbanicity |  |  |  |
| Med-large city | 1 |  |  |
| Suburb | 0.94 | 0.79-1.12 | 0.478 |
| Unincorporated area | 0.83 | 0.67-1.04 | 0.105 |
| Small city or town | **0.77** | **0.63-0.93** | **0.008** |
| Smaller areas or country | **0.64** | **0.51-0.79** | **<0.001** |
| Household size | 0.95 | 0.90-1.00 | 0.052 |
| Feels afraid in neighborhood | 1.06 | 0.89-1.26 | 0.539 |
| Survey year |  |  |  |
| 1998 | 1 |  |  |
| 2002 | 1.12 | 0.93-1.35 | 0.244 |
| 2012 | **0.62** | **0.53-0.74** | **<0.001** |
| 2016 | **0.52** | **0.44-0.63** | **<0.001** |

*Note.* N=7094. For odds ratios, 1 indicates the reference category.
